# Development and Performance Evaluation of a Novel Ancestry Informative DIP Panel for Continental Origin Inference

**DOI:** 10.3389/fgene.2021.801275

**Published:** 2022-02-17

**Authors:** Yongsong Zhou, Xiaoye Jin, Buling Wu, Bofeng Zhu

**Affiliations:** ^1^ Shenzhen Stomatology Hospital (Pingshan), Southern Medical University, Shenzhen, China; ^2^ Guangzhou Key Laboratory of Forensic Multi-Omics for Precision Identification, School of Forensic Medicine, Southern Medical University, Guangzhou, China; ^3^ School of Forensic Medicine, Guizhou Medical University, Guiyang, China; ^4^ Key Laboratory of Shaanxi Province for Craniofacial Precision Medicine Research, College of Stomatology, Xi’an Jiaotong University, Xi’an, China; ^5^ Clinical Research Center of Shaanxi Province for Dental and Maxillofacial Diseases, College of Stomatology, Xi’an Jiaotong University, Xi’an, China

**Keywords:** ancestry informative marker, deletion/insertion polymorphism, AIDIP, forensic ancestry analysis, Eastern Han

## Abstract

Ancestry informative markers (AIMs) are useful to infer individual biogeographical ancestry and to estimate admixture proportions of admixed populations or individuals. Although a growing number of AIM panels for forensic ancestry origin analyses were developed, they may not efficiently infer the ancestry origins of most populations in China. In this study, a set of 52 ancestry informative deletion/insertion polymorphisms (AIDIPs) were selected with the aim of effectively differentiate continental and partial Chinese populations. All of the selected markers were successfully incorporated into a single multiplex PCR panel, which could be conveniently and efficiently detected on capillary electrophoresis platforms. Genetic distributions of the same 50 AIDIPs in different continental populations revealed that most loci showed high genetic differentiations between East Asian populations and other continental populations. Population genetic analyses of different continental populations indicated that these 50 AIDIPs could clearly discriminate East Asian, European, and African populations. In addition, the 52 AIDIPs also exhibited relatively high cumulative discrimination power in the Eastern Han population, which could be used as a supplementary tool for forensic investigation. Furthermore, the Eastern Han population showed close genetic relationships with East Asian populations and high ancestral components from East Asian populations. In the future, we need to investigate genetic distributions of these 52 AIDIPs in Chinese Han populations in different regions and other ethnic groups, and further evaluate the power of these loci to differentiate different Chinese populations.

## Introduction

Ancestry informative markers (AIMs) refer to genetic variations that exhibit high allelic frequency divergences between different ancestral populations (Phillips et al., 2007). AIMs are useful to infer individual biogeographical ancestry and to estimate admixture proportions of admixed populations or individuals. In the last decade, as a new supplementary test, forensic ancestry information analysis provides much valuable information for forensic investigative applications and other forensic fields (Phillips, 2015; Phillips and de la Puente, 2021). Most recently, a growing number of AIM panels to estimate ancestry origin of continental and sub-continental populations (Santos et al., 2016a; Wei et al., 2016; Carvalho Gontijo et al., 2020; Xavier et al., 2020) or to distinguish population structure of Asian or Chinese populations (Sun et al., 2016; Jin et al., 2019; Qu et al., 2019) were developed by forensic researchers from abroad and in China, respectively. However, the capacity of these panels to effectively infer the ancestry origins of other populations in China may not be competent enough. Furthermore, large-scale and representative population genetic data are the key element of forensic assay development and application. Unfortunately, AIM reference population data in most Chinese populations are still undeveloped to date, which limit population-specific marker selections to some extent. Accordingly, we need to investigate genetic distributions of more AIMs in Chinese populations. These data can not only enrich the genetic information resources of Chinese population, but also facilitate the screening of population specific molecular markers.

Deletion/insertion polymorphisms (DIPs) are one type of genetic variations that arise from random deletion or insertion of DNA fragments (Weber et al., 2002). This kind of polymorphism exhibits unique characteristics as AIMs: (i) wide distributions in the human genome; (ii) with low mutation rates; (iii) the frequencies of alleles varies greatly between populations; and (iv) can be easily detected by multiplex PCR and capillary electrophoresis platform (Santos et al., 2010; Li et al., 2012; LaRue et al., 2014). In recent years, DIPs received a large amount of attention from forensic geneticists. A set of DIP panels for various forensic purposes have been constructed. For example, Chen et al. developed a multiplex panel of autosomal DIPs for forensic identity testing (Chen et al., 2019); Lan et al. presented a multiplex system of 39 ancestry informative DIPs (AIDIPs) for forensic ancestry origins of three different continental populations (Lan et al., 2019); Chen et al. also constructed a novel multiplex system that could detect 38 X-chromosome DIPs to assist in individual identification and paternity testing (Chen et al., 2021). Collectively, the DIPs showed great application values in forensic research.

In this study, we firstly selected 52 AIDIPs for ancestry origin predictions of different continental populations based on the 1,000 Genome Project (Genomes Project et al., 2015) and previous studies (Mills et al., 2006; Pereira et al., 2009; Santos et al., 2010; Pereira et al., 2012b). Secondly, we evaluated the efficiencies of these AIDIPs for dissecting continental population structure. At the same time, a multiplex panel of these 52 AIDIPs was developed on the basis of capillary electrophoresis platform. Next, genetic distributions and forensic statistical parameters of these 52 AIDIPs in Eastern Han population were assessed. Finally, ancestral components of Eastern Han population were explored in comparison with continental populations.

## Materials and Methods

### AIDIPs Selection and Development of the Multiplex Panel

We aim to construct a multiplex PCR assay of 52 AIDIPs based on the capillary electrophoresis platform for forensic individual biogeographic ancestry inference and population genetic structure and background analyses. A batch of 52 AIDIPs located on autosomal chromosome were selected; they showed high allele frequency divergences among European, East Asian, and African populations, which were confirmed by previous studies (Mills et al., 2006; Pereira et al., 2009; Santos et al., 2010; Pereira et al., 2012b). AIDIPs selection criteria were consistent with Lan et al. (2019). We screened 52 biallelic DIP genetic markers that performed the following requirements: (i) all DIP markers were selected from the autosomes; (ii) variable size of deletion/insertion fragments ranged from 2 to 20 bp; (iii) allele frequency differentials ≥0.2 between at least two continental populations; and (iv) no departures from Hardy–Weinberg equilibrium (HWE) in any continental population. The detailed genomic information and reference sequences of these selected AIDIP loci were obtained from dbSNP (http://www.ncbi.nlm.nih.gov/SNP/). The primer design and multiplex assay construction of 52 AIDIP loci proceeded based on the workflows described by Pereira et al. (2012a; 2012b). Primers were designed by the Primer Premier 5.0 software according to the following two main principles: the T_m_ value was close to 65°C and the amplicon sizes varied from 60 to 250 bp. Potential primer dimers and hairpin structures were evaluated by AutoDimerv1 software. Subsequently, all markers were assigned and labeled by four different fluorescent dyes (FAM, HEX, TAMRA, and ROX), respectively, and all of the primers were synthesized by Sangon Biotech (Sangon Biotech Co., Ltd., Shanghai, China).

### Multiplex Amplification and AIDIP Genotyping

The PCR reaction of the 52 AIDIPs was finally optimized to amplify in a single tube with the 25-μl reaction volume. The reaction system composed of 5 μl of primers, 10 μl of reaction mix (AGCU Biotech Co., Ltd., Wuxi, China), 1 μl of template DNA, 1 μl of polymerase (5 U/μl, Takara Biomedical Technology Co., Ltd., Beijing, China), and 8 μl of sdH_2_O. The PCR cycling conditions were as follows: 95°C for 5 min; 30 cycles of 94°C for 15 s, 60°C for 50 s, and 62°C for 55 s; and a final extension at 70°C for 20 min.

For capillary electrophoresis, 1 μl of amplification products were added to 12.5 μl of loading mixtures, which consisted of 12 μl of deionized Hi-Di^®^ formamide (Thermo Fisher Scientific, Waltham, MA United States) and 0.5 μl of AGCU SIZ-500 internal size standard (AGCU Biotech). Detection and separation for 52 AIDIPs were performed on 3500xL Genetic Analyzers (Thermo Fisher Scientific) under default injection conditions. The raw data were genotyped with GeneMapper^®^ ID-X v1.5 software (Thermo Fisher Scientific).

### Ethics Statement, Population Sample Collection, and Genomic DNA Extraction

Buccal samples were obtained from the volunteers with written informed consents for the above-mentioned research purposes, approved by the Ethics Committee of Xi’an Jiaotong University, China (No. XJTULAC 2013). Buccal samples stored on FTA™ cards (GE Healthcare, Buckinghamshire, United Kingdom) were collected from 345 unrelated healthy Han individuals who lived in eastern China for more than three generations, including 200 individuals living in Wuxi city, Jiangsu Province and 145 individuals living in Hangzhou city, Zhejiang Province, China. The genomic DNA was extracted and quantified using Chelex^®^ 100 resin-based method (Phillips et al., 2012) and the Applied Biosystems^®^ 7,500 Real-Time PCR System (Thermo Fisher Scientific), respectively. Genomic DNA was diluted to 1 ng per microliter with Tris-EDTA buffer and stored at −20°C for later use.

### Statistical Analysis

Firstly, we assessed allelic frequency distributions of the same 50 AIDIPs in different continental populations. A heatmap of deletion allelic frequencies of 50 AIDIPs in African, American, East Asian, European, and South Asian populations was plotted by the pheatmap package v1.0.12 of *R* software v4.1.0. Pairwise fixation index (*F*_*ST*_) and informativeness (*In*) values of 50 AIDIPs among continental populations were calculated by Arlequin software v3.5.1.2 (Excoffier et al., 2007) and Infocalc software version 1.1 (Rosenberg et al., 2003), respectively. Then, the *F*_*ST*_ and *In* values were graphically displayed by the TBtools software v1.09861 (Chen et al., 2020) and ggplot2 package version 3.3.0 of *R* software, respectively. Population-specific divergences (PSDs) of 50 AIDIPs in each continental population were estimated by a previous report (Phillips, 2015). Next, the performance of these AIDIPs for inferring ancestry origins of continental populations was evaluated by the following methods. Principal component analysis (PCA) of five continental populations was conducted using the Plink software version 1.9 (Chang et al., 2015), and then a scatter plot of these population levels was drawn by the ggplot2 package. Genetic structure of these continental populations was explored by the Admixture software version 1.3 at *K* = 2–7 (Alexander et al., 2009). Thorough analyses of different continental populations were performed by the *Snipper* online tool v2.5 (http://mathgene.usc.es/snipper/) based on 50 AIDIPs.

For the Eastern Han population, allelic frequencies, forensic statistical parameters, HWE tests, and linkage disequilibrium analyses of the 52 AIDIPs were estimated by the STRAF online tool v1.0.5 (Gouy and Zieger, 2017). PCA of Eastern Han and continental populations was also conducted by Plink and ggplot2 packages. Genetic structure of Eastern Han population was assessed by the Admixture software. Different continental populations were viewed as training sets and the Eastern Han population was viewed as the testing samples, and then ancestry origin analyses of Eastern Han population were assessed by the *Snipper*.

## Results and Discussion

### Development of a Novel AIDIPs Multiplex Assay

**FIGURE 1:**
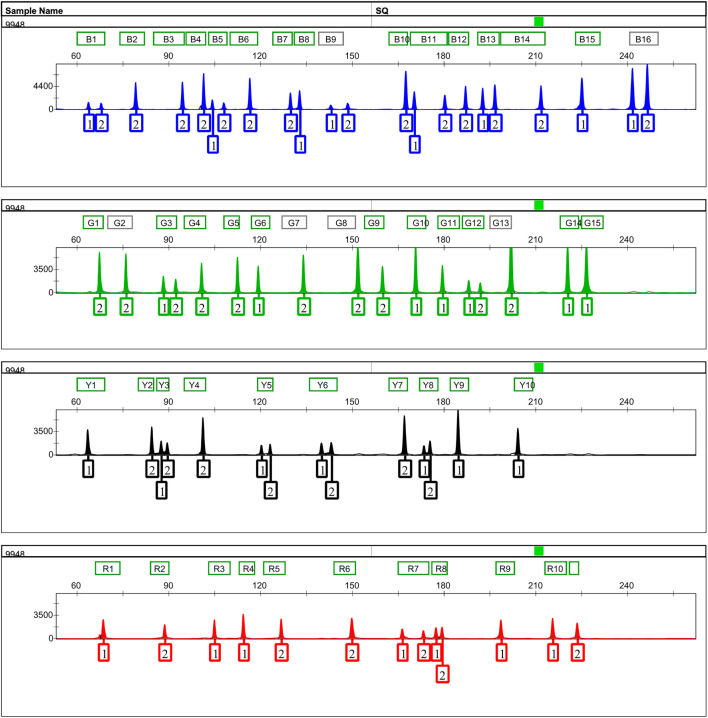
Representative 500 pg of Control DNA 9948 profile amplified with the 52-AIDIP panel for 25-μl reaction volumes. Five hundred picograms of Control DNA 9948 was amplified with the developed panel for 30 cycles. One microliter of PCR product added into 12.5 μl of loading mixtures (12 μl Hi-Di formamide +0.5 μl SIZ-500 Size Standard) was electrophoresed on a 3500xL Genetic Analyzer using the default injection conditions.

An informative and applicable AIDIPs multiplex assay was developed for simultaneous genotyping of 52 AIDIP loci on the basis of capillary electrophoresis platform. The 52 AIDIP loci were laid out in blue (FAM), green (HEX), yellow (TAMRA), and red (ROX) dye channels according to dye color and expected amplicon size (Table 1). The size of amplicons varied from 63 bp at the deletion alleles of loci rs3092383, rs10549914, and rs11576045 to 246 bp at the insertion allele of rs3028297 locus. Generally, full profiles were obtained when various amounts of template DNA (0.2–10 ng) were added, during the testing of the Eastern Han population. However, the optimal concentration of template DNA for this multiplex assay is 0.5–5 ng in a 25-μl PCR final volume. When the amounts of inputted DNA were above 5 ng or below 0.5 ng, the intra-locus and/or intra-color imbalance were randomly observed. As illustrated in Figure 1, a complete genotyping profile was obtained when 500 pg of Control DNA 9948 (Promega Corporation, Madison, WI, United States) was added into a 25-μl reaction volume. Compared with AIDIP panels previously reported (Santos et al., 2010; Pereira et al., 2012b; Sun et al., 2016; Lan et al., 2019), the assay developed in this study involved a higher number of AIDIP loci in a single PCR reaction system. More AIDIPs might be more beneficial to discriminate Chinese populations than these reported panels, which remained to be investigated further.

**TABLE 1:** General information of the 52 AIDIP loci. The numbers 1 and 2 in the “Genotype of 9948” column represent deletion and insertion of nucleotides, respectively.

ID number	Internal code	rs number	Chromosome	Position (GRCh38)	Alleles described in dbSNP	Genotype of 9948	Range of amplicon size (bp)	Fluorescent labels
1	B1	rs3092383	Chr20	46848769	-/AACA	1,2	60–69	FAM
2	B2	rs140864	Chr1	35926061	-/TTC	2	74–82	FAM
3	B3	rs3033053	Chr14	42085293	-/TCAGCAG	2	85–95	FAM
4	B4	rs72375069	Chr3	27386331	-/AATT	2	95.6–102	FAM
5	B5	rs140498743	Chr3	139513672	-/TGTC	1,2	103–109	FAM
6	B6	rs67205569	Chr10	93181810	-/TTGAC	2	110–119	FAM
7	B7	rs74499778	Chr11	130071487	-/AGCT	2	124–130	FAM
8	B8	rs139220746	Chr2	199340972	-/TATC	1	131–137.5	FAM
9	B9	rs10668859	Chr19	266759	-/GAAAG	1,2	139–147	FAM
10	B10	rs140847	Chr9	12617325	-/CGTT	2	162–168	FAM
11	B11	rs16711	Chr17	20179106	-/TTTCTTCCTA	1,2	169–181	FAM
12	B12	rs149676649	Chr5	28495279	-/GATT	2	181.5–188	FAM
13	B13	rs57237250	Chr6	109941799	-/GAGT	1,2	191–198	FAM
14	B14	rs2308163	Chr14	57583363	-/TGAT	2	198.5–213	FAM
15	B15	rs16438	Chr20	25297829	-/CCCAC/CCCCA	1	223–231	FAM
16	B16	rs3028297	Chr9	104604012	-/GCTAA/CTAA	1,2	240.72–250	FAM
17	G1	rs10549914	Chr17	5425659	-/TTTA	2	62–68.5	HEX
18	G2	rs10581451	Chr8	72942426	-/TGAG	2	70–78	HEX
19	G3	rs67934853	Chr2	74716761	-/TAAC	1,2	86–92.5	HEX
20	G4	rs3831920	Chr1	1292285	-/CTCA	2	95–102	HEX
21	G5	rs1160852	Chr6	137024720	-/TT	2	108–113	HEX
22	G6	rs25630	Chr6	14734110	-/AG	1	117–123	HEX
23	G7	rs2307998	Chr5	7814232	-/GGA	2	127–135	HEX
24	G8	rs138123572	Chr15	72493894	-/TGAC	2	142–151	HEX
25	G9	rs3839049	Chr2	26254260	-/ACT	2	154–160	HEX
26	G10	rs2307840	Chr1	35633488	-/GT	1	168–174	HEX
27	G11	rs35779249	Chr13	43390341	-/TAA	1	178–185	HEX
28	G12	rs1305047	Chr17	16181674	-/CACA	1,2	186–193	HEX
29	G13	rs66693708	Chr12	77004626	-/TAAG	2	195–202	HEX
30	G14	rs2308036	Chr15	64914812	-/CC	1	218–224	HEX
31	G15	rs3074939	Chr21	42002321	-/CAGT	1	225–232	HEX
32	Y1	rs11576045	Chr12	111361720	-/ACA	1	60–69	TAMRA
33	Y2	rs3217613	Chr15	84932992	-/ATA	2	80–85	TAMRA
34	Y3	rs3059936	Chr11	112701065	-/AT	1,2	86–90	TAMRA
35	Y4	rs3840274	Chr4	68494125	-/CTCA	2	95–102	TAMRA
36	Y5	rs3840614	Chr7	78029712	-/TTC	1,2	119–124	TAMRA
37	Y6	rs3033100	Chr4	140872558	-/CAG	1,2	136–145	TAMRA
38	Y7	rs3838001	Chr20	63684046	-/CAA	2	162–168	TAMRA
39	Y8	rs3049003	Chr7	6660366	-/AT	1,2	172–178	TAMRA
40	Y9	rs3051160	Chr10	100927312	-/TG	1	182–188	TAMRA
41	Y10	rs5796380	Chr12	10124046	-/AAG	1	203–209	TAMRA
42	R1	rs5783058	Chr10	8742655	-/TGTT	1	66–74	ROX
43	R2	rs3216128	Chr21	42559334	-/AGA	2	84–90	ROX
44	R3	rs5822884	Chr18	5980141	-/TAGT	1	103–110	ROX
45	R4	rs3053514	Chr21	28691710	-/TAC	1	113–118	ROX
46	R5	rs3840019	Chr15	65752777	-/AATT	2	121–128	ROX
47	R6	rs1610951	Chr5	109664135	-/CCAA	2	144–151	ROX
48	R7	rs1305057	Chr5	57319423	-/TGTTTCA	1,2	165–175	ROX
49	R8	rs3073179	Chr11	18237493	-/AT	1,2	176–181	ROX
50	R9	rs2307727	Chr2	135675653	-/TT	1	197–203	ROX
51	R10	rs5891726	Chr8	59361584	-/TACT	1	213–220	ROX
52	R11	rs5824539	Chr18	44391691	-/TA	2	221–224	ROX

### Genetic Distributions of Selected AIDIPs in Different Continental Populations

**FIGURE 2:**
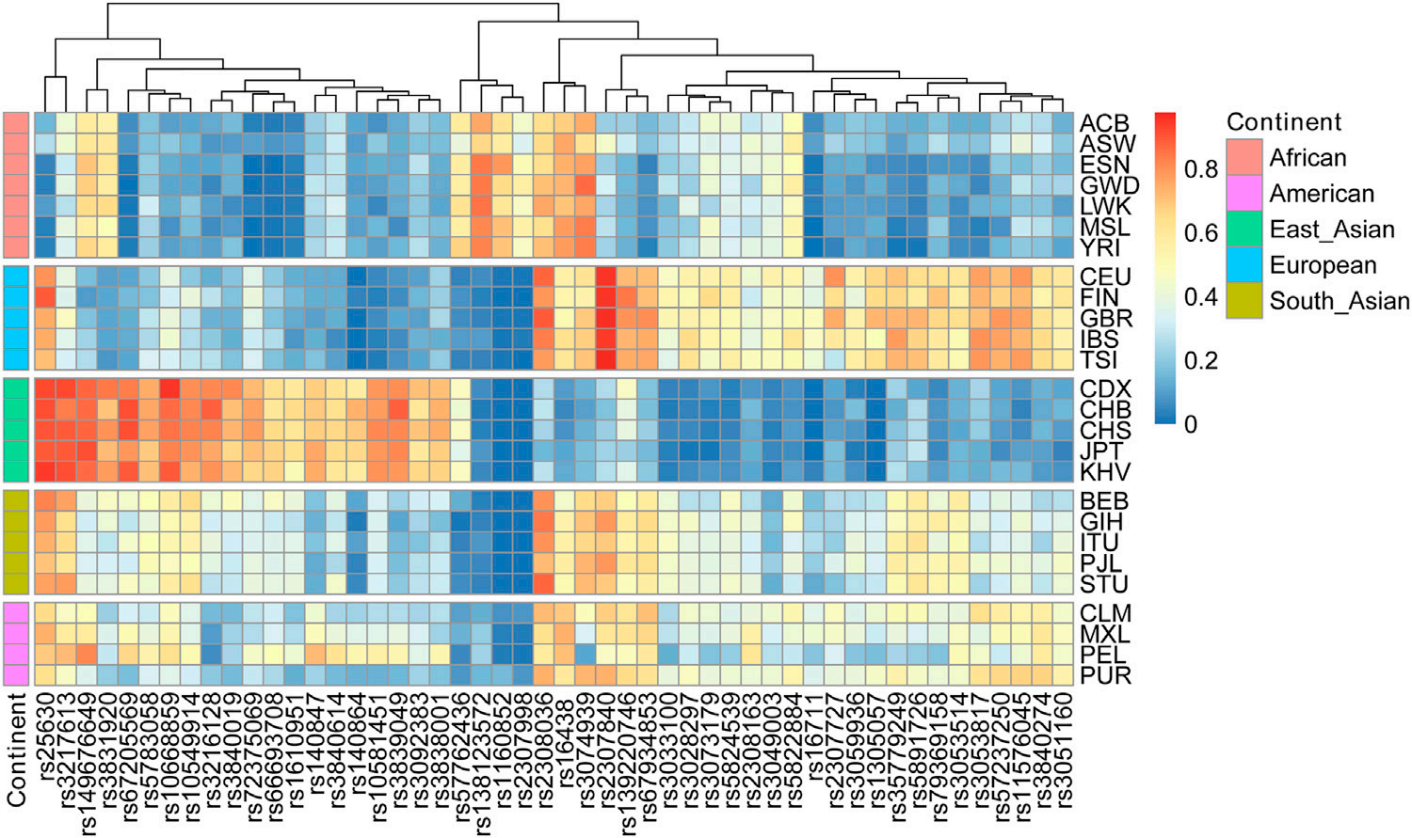
Heatmap of deletion allelic frequencies of 50 ancestry informative DIPs in different continental populations.

Although 52 AIDIP loci were selected and successfully incorporated into the novel assay for ancestry origin inference, the population data of rs3033053 and rs1305047 loci were not available in the 1,000 Genome Project. Thus, genetic data of the same 50 AIDIPs were assessed in five different continental populations. To visually display the analytical results, the distributions of deletion allele frequencies of the 50 AIDIPs are shown by a heatmap. As shown in Figure 2, the allelic frequencies for the vast majority of these selected AIDIPs varied greatly among different populations. For example, rs67205569, rs10668859, rs149676649, rs3839049, rs3217613, and rs3216128 loci displayed relatively high frequencies in the East Asian populations. It is important to note that 46 AIDIP loci showed almost completely opposite allelic frequency distributions between East Asian and European populations with the exception of rs25630, rs138123572, rs1160852, and rs2307998 loci. It seems to imply that these loci were of considerable potency to distinguish East Asian populations from European populations. Furthermore, we also found that rs25630, rs3217613, rs138123572, rs1160852, and rs2307998 loci exhibited significant allele frequency differences between African and non-African populations: American, European, South Asian, and East Asian populations. However, the differences of allele distributions between American and European/South Asian populations were relatively small for most loci.

**FIGURE 3:**
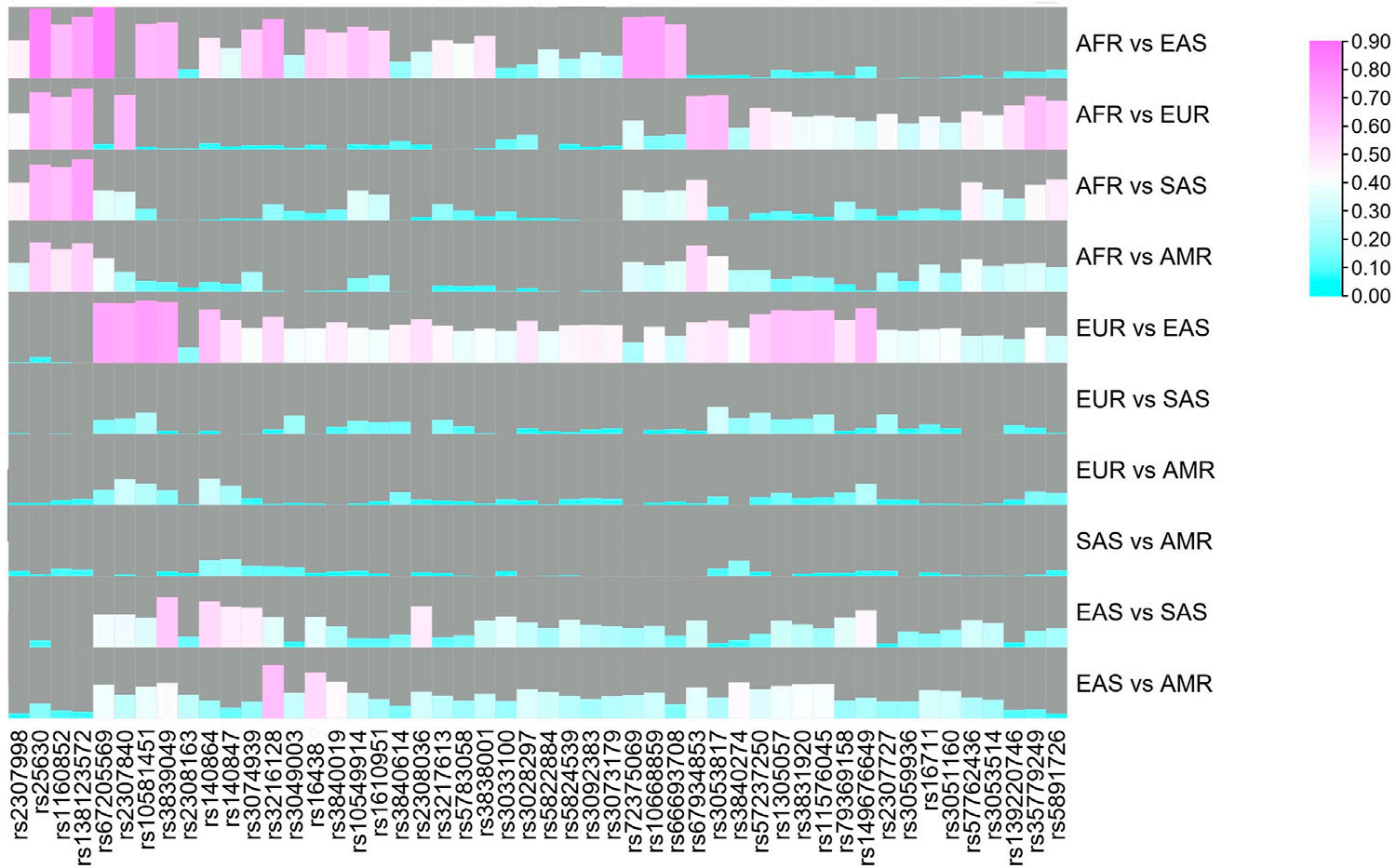
Pairwise *F*_*ST*_ values of five continental populations for 50 ancestry informative DIPs.

To reveal genetic divergences of these AIDIPs among different continental populations better, pairwise *F*_*ST*_ values were also calculated, as shown in Figure 3. Results revealed that most loci showed relatively high *F*_*ST*_ values between East Asian and other continental populations, especially between East Asians and Europeans, whereas most loci showed low *F*_*ST*_ values between American and European/South Asian populations. *In* is commonly used to evaluate the ancestral information of genetic markers in different populations (Phillips, 2015). Hence, the pairwise *In* values of these 50 AIDIPs were also estimated. Similar to *F*_*ST*_ values, most loci showed high *In* values between East Asian and other continental populations (Supplementary Figure 1). Shriver et al. stated that the developed AIM panel should possess balance differentiation efficiencies among each population, which could bring little bias into ancestral components of admixed individuals (Shriver et al., 2004). Therefore, we assessed the cumulative PSD values of 50 AIDIPs in the five continental populations. Results demonstrated that these 50 loci showed the highest cumulative PSD values in the East Asian population, followed by African, European, South Asian, and American populations (Supplementary Figure 2). In this study, to infer ancestry origins of East Asian populations more accurately, we selected AIDIP loci that showed high genetic variations between East Asian and other continental populations, resulting in higher cumulative PSD values in East Asian populations. In addition, we found that 50 AIDIP loci also showed relatively high cumulative PSD values in European and African populations. Nonetheless, relatively low cumulative PSD values of these 50 loci in South Asian and American populations suggested that they might not be suitable for ancestry origin analyses of these two intercontinental populations.

### Ancestry Resolutions of the Developed AIDIP Panel for Continental Populations

**FIGURE 4:**
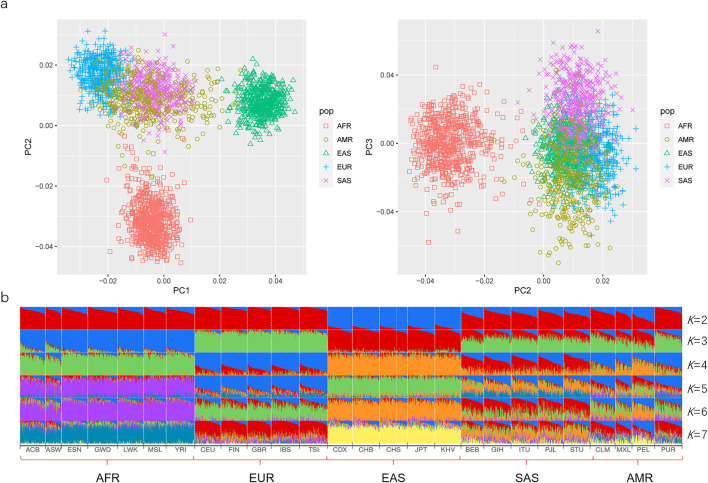
Ancestry origin analyses of different continental populations based on 50 ancestry informative DIPs. **(A)** PCA of different continental populations at PC1, PC2, and PC3. **(B)** Population genetic structure analyses of different continental populations at *K* = 2–7.

Here, the PCA was primarily conducted on the basis of the same 50 AIDIPs to evaluate the capacity of the developed AIDIP assay to differentiate continental populations. Results of the PCA analysis for the five continental populations are shown in Figure 4A. At PC1, African, European, and East Asian individuals formed three population clusters, respectively, and they could be clearly separated from each other. At PC3, some South Asian and American individuals could be differentiated from other continental populations. Subsequently, the genetic structure of these continental populations was also explored. The results with *K* ranging from two to seven are presented in Figure 4B. At *K* = 2, five East Asian populations exhibited high blue components and could be discriminated from other populations. As *K* becomes 3, African, European, and East Asian populations showed their distinct ancestral components, respectively. Moreover, American and South Asian populations showed similar ancestral component distributions. When *K* increased to 4, South Asian populations could be separated from other populations. No more significant changes in population structure were observed from the bar plot when the *K* values were greater than 4. These results demonstrated that the novel AIDIP panel could clearly differentiate African, European, and East Asian populations. The capacity of this assay to differentiate continental populations is similar to those of previously reported panels (Santos et al., 2010; Pereira et al., 2012b; Lan et al., 2019). Nevertheless, unlike the weaker capacity of the 46-AIM-InDels panel to differentiate the East Asian population (Pereira et al., 2012b), the current AIDIP panel revealed an excellent characteristic to estimate the ancestry information of East Asians.

The *Snipper* online tool was developed to infer ancestry origins of populations by the Bayesian method (Santos et al., 2016b). Therefore, we further evaluated ancestry resolutions of 50 AIDIPs for continental populations by the *Snipper*. Results indicated that most individuals from African, European, East Asian, and South Asian populations could be classified into correct continental origins, whereas some individuals from American populations were classified into European and South Asian populations (Supplementary Figure 3). Admixed genetic background of American populations went against their ancestry origin inferences (Genomes Project et al., 2015). In addition, relatively few American-specific genetic markers in the extant panel might also lead to this result. Even so, obtained results revealed that these 50 AIDIPs could be utilized to differentiate African, European, and East Asian populations well.

### Allelic Frequencies and Forensic Statistical Parameters of 52 AIDIPs in Eastern Han Population

HWE tests of 52 AIDIPs in the Eastern Han population are given in Supplementary Table 1. No loci were observed to deviate from HWE after applying Bonferroni correction (*p* = 0.05/52 = 0.00096). Linkage disequilibrium (LD) analyses of pairwise AIDIPs in the Eastern Han population are listed in Supplementary Table 2. The results showed that a significant association between rs2307840 and rs140864 loci was revealed, even after applying Bonferroni correction (*p* = 0.05/1,326 = 0.000037). LD between the two loci in the Eastern Han population may be caused by genetic linkage because both of them are located on chromosome one and just 292,573 bp apart from each other. The locus rs140864 exhibited a little more excellent characteristic of forensic statistical parameters, therefore, it was preferentially selected for further data analysis in Eastern Han population. Furthermore, to better understand the associations among loci in different population groups, further evaluations containing more populations and lager sample sizes need to be investigated.

**FIGURE 5:**
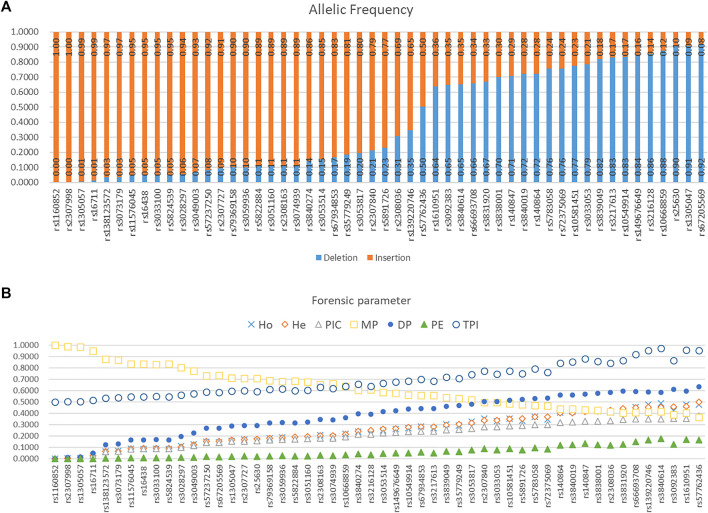
Allelic frequencies and forensic parameters of 52 ancestry informative DIPs in an Eastern Han population. **(A)** Allelic frequency. **(B)** Forensic parameter.

Allelic frequencies of 52 AIDIPs in the Eastern Han population are presented in Figure 5A and Supplementary Table 1. Deletion allelic frequencies of these loci ranged from 0.0000 to 0.9159. We also calculated forensic parameters of these loci in Eastern Han population, as given in Figure 5B and Supplementary Table 1. Mean observed heterozygosity (Ho), expected heterozygosity (He), polymorphism information content (PIC), match probability (MP), discrimination power (DP), power of exclusion (PE), and typical paternity index (TPI) of 52 AIDIPs in Eastern Han population were 0.2491, 0.2527, 0.2107, 0.6215, 0.3785, 0.0594, and 0.6910, respectively. Cumulative DP and PE of these loci in the Eastern Han population were 0.999 999 999 9977646 and 0.9619, respectively. As expected, these loci exhibited relatively low genetic diversities in the Eastern Han population. Even so, relatively high cumulative DP indicated that these loci could be viewed as a supplementary tool for forensic identity testing in the Eastern Han population.

### Ancestry Component Dissections of Eastern Han Populations by 50 AIDIPs

**FIGURE 6:**
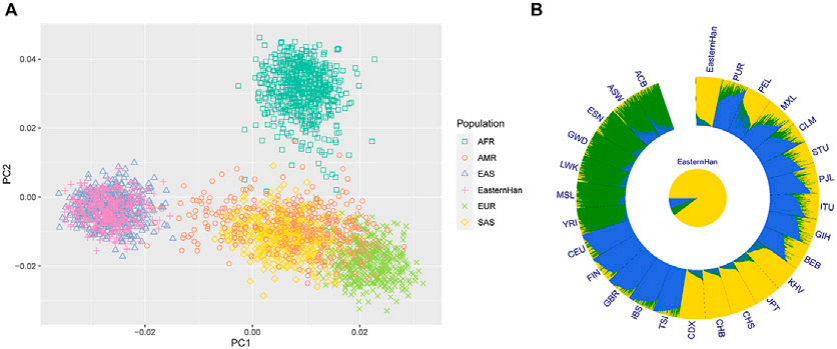
Ancestry component analyses of the studied Eastern Han populations in comparisons with other continental populations. **(A)** PCA of Eastern Han and continental populations at PC1 and PC2. **(B)** Population genetic structure analyses of Eastern Han and continental populations at *K* = 3.

Based on the raw data of 50 AIDIPs, PCA of Eastern Han and continental populations was conducted. We found that the studied Eastern Han individuals were predominately superimposed on the East Asian individual cluster located on the right part of the plot (Figure 6A). Ancestral components of the Eastern Han populations were also assessed in comparisons to five continental populations, as presented in Figure 6B. The studied Eastern Han population displayed high ancestral components from East Asian populations. Subsequently, we treated five continental populations as training set and Eastern Han population as unknown population and explored the power of these AIDIPs to infer ancestral origins of Eastern Han population by the *Snipper*. The obtained results revealed that all Eastern Han individuals could be categorized into East Asian population, implying that these AIDIPs could perform ancestry origin analyses of Eastern Han population well. Besides, these results also reflected that the studied Eastern Han population had intimate genetic relationships with East Asian populations.

Lang et al. assessed genetic structure of Eastern Han population by 27 Y-STRs and 143 Y-SNPs and found that the Han populations showed closer genetic affinities with East Asian populations than South Asian populations. Furthermore, they also pointed out that genetic differentiations between Southern Han and Northern Han populations were observed (Lang et al., 2019). Lu et al. investigated genetic distributions of 17 autosomal STRs in an Eastern Han population (Jiangsu Han) and they found that the Han population showed low genetic divergences with Hubei Han populations (Lu et al., 2019). Chiang et al. conducted a comprehensive analysis of genetic variations in Chinese Han populations and found an east–west differentiation among Han populations except for a known south–north cline (Chiang et al., 2018). Moreover, Li et al. exploited the genetic landscape of Chinese Han populations based on the mitochondria DNA and revealed that genetic divergences among Han populations residing in different river systems existed (Li et al., 2019). On this basis, we speculated that genetic substructure potentially existed among different Han populations in China. Consequently, we intend to investigate genetic polymorphism distributions of selected 52 AIDIPs in Han populations from different regions. Those studies can not only depict the genetic architecture of different Han Chinese populations, but also contribute to screen region-specific genetic markers. Moreover, due to the large allele frequency differences between European and East Asian populations of these AIDIP loci selected in the present study, next we intend to explore the capacity of this novel assay to infer the ancestral origins of groups with admixed Eurasian ancestry in China.

## Conclusion

In summary, we developed a multiplex PCR panel for ancestry origin predictions of different continental populations that contained 52 AIDIP loci. Most loci out of these 52 AIDIPs showed high genetic divergences between East Asian and non-East Asian populations. We also demonstrated that this AIDIP panel could be employed for inferring biogeographical origins of continental populations, specifically for East Asian, African, and European populations. In addition, these 52 AIDIP loci also showed relatively high application values for forensic identity testing in the Eastern Han population. For ancestral component analysis of the Eastern Han population, the novel panel could accurately estimate its close genetic affinities and high ancestral components with East Asian populations. In the future, we need to assess genetic distributions of the 52 AIDIPs in other populations from different regions to unveil genetic portraits of these populations. Only in this way could the performance of the developed panel to infer sub-populations and estimate inter-ethnic admixture proportions be completely understood.

## Data Availability

The datasets presented in this article are not readily available to maintain the participants privacy. Requests to access the datasets should be directed to the corresponding author, BZ.

## Supplementary Material

Supplementary Figure 1 Pairwise *In* values of five continental populations for 50 ancestry informative DIPs.

Supplementary Figure 2 Cumulative PSD values of 50 ancestry informative DIPs in different continental populations.

Supplementary Figure 3 Ancestry origin analyses of different continental populations by the *Snipper* 2.5 online tool.

## References

[B1] Alexander D. H. Novembre J. Lange K. (2009). Fast Model-Based Estimation of Ancestry in Unrelated Individuals. Genome Res. 19 (9), 1655–1664. 10.1101/gr.094052.109 19648217 PMC2752134

[B2] Carvalho Gontijo C. Porras-Hurtado L. G. Freire-Aradas A. Fondevila M. Santos C. Salas A. (2020). PIMA: A Population Informative Multiplex for the Americas. Forensic Sci. Int. Genet. 44, 102200. 10.1016/j.fsigen.2019.102200 31760353

[B3] Chang C. C. Chow C. C. Tellier L. C. Vattikuti S. Purcell S. M. Lee J. J. (2015). Second-generation PLINK: Rising to the challenge of Larger and Richer Datasets. GigaSci. 4, 7. 10.1186/s13742-015-0047-8 PMC4342193 25722852

[B4] Chen C. Chen H. Zhang Y. Thomas H. R. Frank M. H. He Y. (2020). TBtools: An Integrative Toolkit Developed for Interactive Analyses of Big Biological Data. Molecular Plant 13 (8), 1194–1202. 10.1016/j.molp.2020.06.009 32585190

[B5] Chen L. Du W. Wu W. Yu A. Pan X. Feng P. (2019). Developmental Validation of a Novel Six-Dye Typing System with 47 A-InDels and 2 Y-InDels. Forensic Sci. Int. Genet. 40, 64–73. 10.1016/j.fsigen.2019.02.009 30776773

[B6] Chen L. Pan X. Wang Y. Du W. Wu W. Tang Z. (2021). Development and Validation of a Forensic Multiplex System with 38 X-InDel Loci. Front. Genet. 12, 670482. 10.3389/fgene.2021.670482 34484288 PMC8416044

[B7] Chiang C. W. K. Mangul S. Robles C. Sankararaman S. (2018). A Comprehensive Map of Genetic Variation in the World's Largest Ethnic Group-Han Chinese. Mol. Biol. Evol. 35 (11), 2736–2750. 10.1093/molbev/msy170 30169787 PMC6693441

[B8] Excoffier L. Laval G. Schneider S. (2007). Arlequin (Version 3.0): an Integrated Software Package for Population Genetics Data Analysis. Evol. Bioinform Online 1, 47–50. 19325852 PMC2658868

[B9] Genomes Project C. Auton A. Brooks L. D. Durbin R. M. Garrison E. P. Kang H. M. (2015). A Global Reference for Human Genetic Variation. Nature 526 (7571), 68–74. 10.1038/nature15393 26432245 PMC4750478

[B10] Gouy A. Zieger M. (2017). STRAF-A Convenient Online Tool for STR Data Evaluation in Forensic Genetics. Forensic Sci. Int. Genet. 30, 148–151. 10.1016/j.fsigen.2017.07.007 28743032

[B11] Jin X.-Y. Wei Y.-Y. Lan Q. Cui W. Chen C. Guo Y.-X. (2019). A Set of Novel SNP Loci for Differentiating continental Populations and Three Chinese Populations. PeerJ 7, e6508. 10.7717/peerj.6508 30956897 PMC6445247

[B12] Lan Q. Shen C. Jin X. Guo Y. Xie T. Chen C. (2019). Distinguishing Three Distinct Biogeographic Regions with an In‐house Developed 39‐AIM‐InDel Panel and Further Admixture Proportion Estimation for Uyghurs. Electrophoresis 40 (11), 1525–1534. 10.1002/elps.201800448 30758063

[B13] Lang M. Liu H. Song F. Qiao X. Ye Y. Ren H. (2019). Forensic Characteristics and Genetic Analysis of Both 27 Y-STRs and 143 Y-SNPs in Eastern Han Chinese Population. Forensic Sci. Int. Genet. 42, e13–e20. 10.1016/j.fsigen.2019.07.011 31353318

[B14] LaRue B. L. Lagacé R. Chang C.-W. Holt A. Hennessy L. Ge J. (2014). Characterization of 114 Insertion/deletion (INDEL) Polymorphisms, and Selection for a Global INDEL Panel for Human Identification. Leg. Med. 16 (1), 26–32. 10.1016/j.legalmed.2013.10.006 24296037

[B15] Li C. Zhang S. Li L. Chen J. Liu Y. Zhao S. (2012). Selection of 29 Highly Informative InDel Markers for Human Identification and Paternity Analysis in Chinese Han Population by the SNPlex Genotyping System. Mol. Biol. Rep. 39 (3), 3143–3152. 10.1007/s11033-011-1080-z 21681421

[B16] Li Y.-C. Ye W.-J. Jiang C.-G. Zeng Z. Tian J.-Y. Yang L.-Q. (2019). River Valleys Shaped the Maternal Genetic Landscape of Han Chinese. Mol. Biol. Evol. 36 (8), 1643–1652. 10.1093/molbev/msz072 31112995

[B17] Lu Y. Sun H.-j. Zhou J.-c. Wu X. (2019). Genetic Polymorphisms, Forensic Efficiency and Phylogenetic Analysis of 17 Autosomal STR Loci in the Han Population of Wuxi, Eastern China. Ann. Hum. Biol. 46 (7-8), 601–605. 10.1080/03014460.2019.1693628 31790285

[B18] Mills R. E. Luttig C. T. Larkins C. E. Beauchamp A. Tsui C. Pittard W. S. (2006). An Initial Map of Insertion and Deletion (INDEL) Variation in the Human Genome. Genome Res. 16 (9), 1182–1190. 10.1101/gr.4565806 16902084 PMC1557762

[B19] Pereira R. Pereira V. Gomes I. Tomas C. Morling N. Amorim A. (2012a). A Method for the Analysis of 32 X Chromosome Insertion Deletion Polymorphisms in a Single PCR. Int. J. Leg. Med. 126 (1), 97–105. 10.1007/s00414-011-0593-2 21717151

[B20] Pereira R. Phillips C. Alves C. Amorim A. Carracedo Á. Gusmão L. (2009). A New Multiplex for Human Identification Using Insertion/deletion Polymorphisms. Electrophoresis 30 (21), 3682–3690. 10.1002/elps.200900274 19862748

[B21] Pereira R. Phillips C. Pinto N. Santos C. Santos S. E. B. d. Amorim A. (2012b). Straightforward Inference of Ancestry and Admixture Proportions through Ancestry-Informative Insertion Deletion Multiplexing. PLoS One 7 (1), e29684. 10.1371/journal.pone.0029684 22272242 PMC3260179

[B22] Phillips C. de la Puente M. (2021). The Analysis of Ancestry with Small-Scale Forensic Panels of Genetic Markers. Emerg. Top. Life Sci. 5 (3), 443–453. 10.1042/ETLS20200327 33949669

[B23] Phillips C. (2015). Forensic Genetic Analysis of Bio-Geographical Ancestry. Forensic Sci. Int. Genet. 18, 49–65. 10.1016/j.fsigen.2015.05.012 26013312

[B24] Phillips C. Salas A. Sánchez J. J. Fondevila M. Gómez-Tato A. Álvarez-Dios J. (2007). Inferring Ancestral Origin Using a Single Multiplex Assay of Ancestry-Informative Marker SNPs. Forensic Sci. Int. Genet. 1 (3-4), 273–280. 10.1016/j.fsigen.2007.06.008 19083773

[B25] Phillips K. McCallum N. Welch L. (2012). A Comparison of Methods for Forensic DNA Extraction: Chelex-100 and the Qiagen DNA Investigator Kit (Manual and Automated). Forensic Sci. Int. Genet. 6 (2), 282–285. 10.1016/j.fsigen.2011.04.018 21703957

[B26] Qu S. Zhu J. Wang Y. Yin L. Lv M. Wang L. (2019). Establishing a Second-Tier Panel of 18 Ancestry Informative Markers to Improve Ancestry Distinctions Among Asian Populations. Forensic Sci. Int. Genet. 41, 159–167. 10.1016/j.fsigen.2019.05.001 31136932

[B27] Rosenberg N. A. Li L. M. Ward R. Pritchard J. K. (2003). Informativeness of Genetic Markers for Inference of Ancestry*. Am. J. Hum. Genet. 73 (6), 1402–1422. 10.1086/380416 14631557 PMC1180403

[B28] Santos C. Phillips C. Fondevila M. Daniel R. van Oorschot R. A. H. Burchard E. G. (2016a). Pacifiplex : an Ancestry-Informative SNP Panel Centred on Australia and the Pacific Region. Forensic Sci. Int. Genet. 20, 71–80. 10.1016/j.fsigen.2015.10.003 26517174

[B29] Santos C. Phillips C. Gomez-Tato A. Alvarez-Dios J. Carracedo Á. Lareu M. V. (2016b). Inference of Ancestry in Forensic Analysis II: Analysis of Genetic Data. Methods Mol. Biol. 1420, 255–285. 10.1007/978-1-4939-3597-0_19 27259745

[B30] Santos N. P. C. Ribeiro-Rodrigues E. M. Ribeiro-Dos-Santos Â. K. C. Pereira R. Gusmão L. Amorim A. (2010). Assessing Individual Interethnic Admixture and Population Substructure Using a 48-Insertion-Deletion (INSEL) Ancestry-Informative Marker (AIM) Panel. Hum. Mutat. 31 (2), 184–190. 10.1002/humu.21159 19953531

[B31] Shriver M. D. Kennedy G. C. Parra E. J. Lawson H. A. Sonpar V. Huang J. (2004). The Genomic Distribution of Population Substructure in Four Populations Using 8,525 Autosomal SNPs. Hum. Genomics 1 (4), 274–286. 10.1186/1479-7364-1-4-274 15588487 PMC3525267

[B32] Sun K. Ye Y. Luo T. Hou Y. (2016). Multi-InDel Analysis for Ancestry Inference of Sub-populations in China. Sci. Rep. 6, 39797. 10.1038/srep39797 28004788 PMC5177877

[B33] Weber J. L. David D. Heil J. Fan Y. Zhao C. Marth G. (2002). Human Diallelic Insertion/deletion Polymorphisms. Am. J. Hum. Genet. 71 (4), 854–862. 10.1086/342727 12205564 PMC378541

[B34] Wei Y.-L. Wei L. Zhao L. Sun Q.-F. Jiang L. Zhang T. (2016). A Single-Tube 27-plex SNP Assay for Estimating Individual Ancestry and Admixture from Three Continents. Int. J. Leg. Med. 130 (1), 27–37. 10.1007/s00414-015-1183-5 25833170

[B35] Xavier C. de la Puente M. Phillips C. Eduardoff M. Heidegger A. Mosquera-Miguel A. (2020). Forensic Evaluation of the Asia Pacific Ancestry-Informative MAPlex Assay. Forensic Sci. Int. Genet. 48, 102344. 10.1016/j.fsigen.2020.102344 32615397

