# Supplementary figures and images for: Development and Performance Evaluation of a Novel Ancestry Informative DIP Panel for Continental Origin Inference
Source: Front Genet. 2022 Feb 17;12:801275. doi: 10.3389/fgene.2021.801275 (PMC8891605; doi:10.3389/fgene.2021.801275)

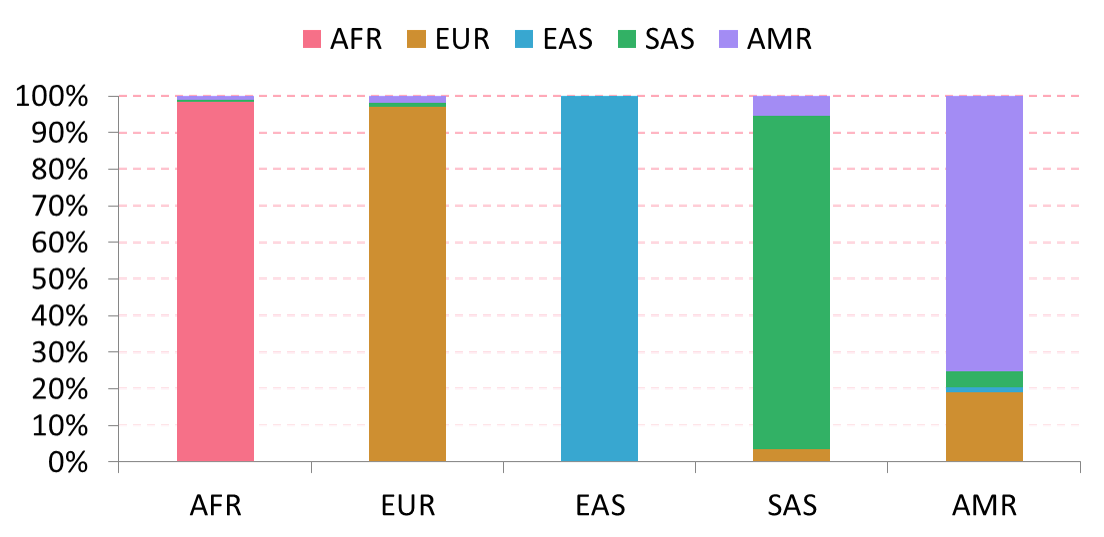

Supplement: Supplementary file 2 [file Image3.TIF]

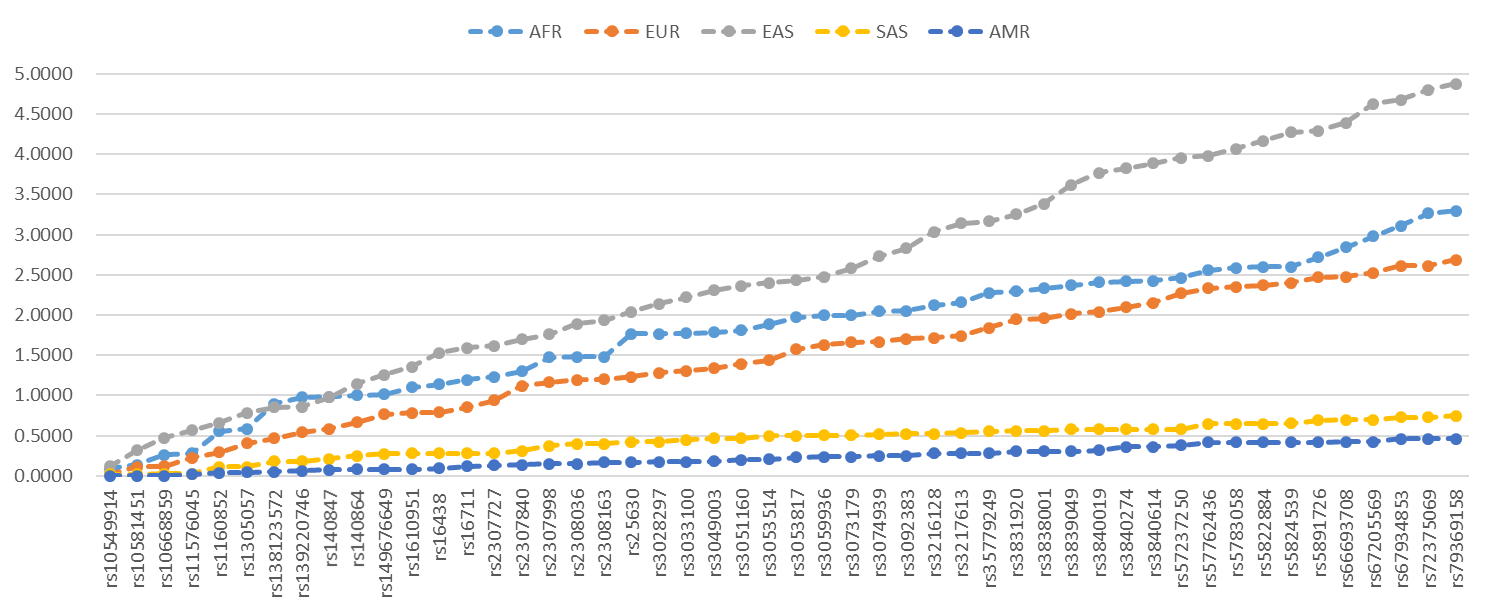

Supplement: Supplementary file 3 [file Image2.TIF]

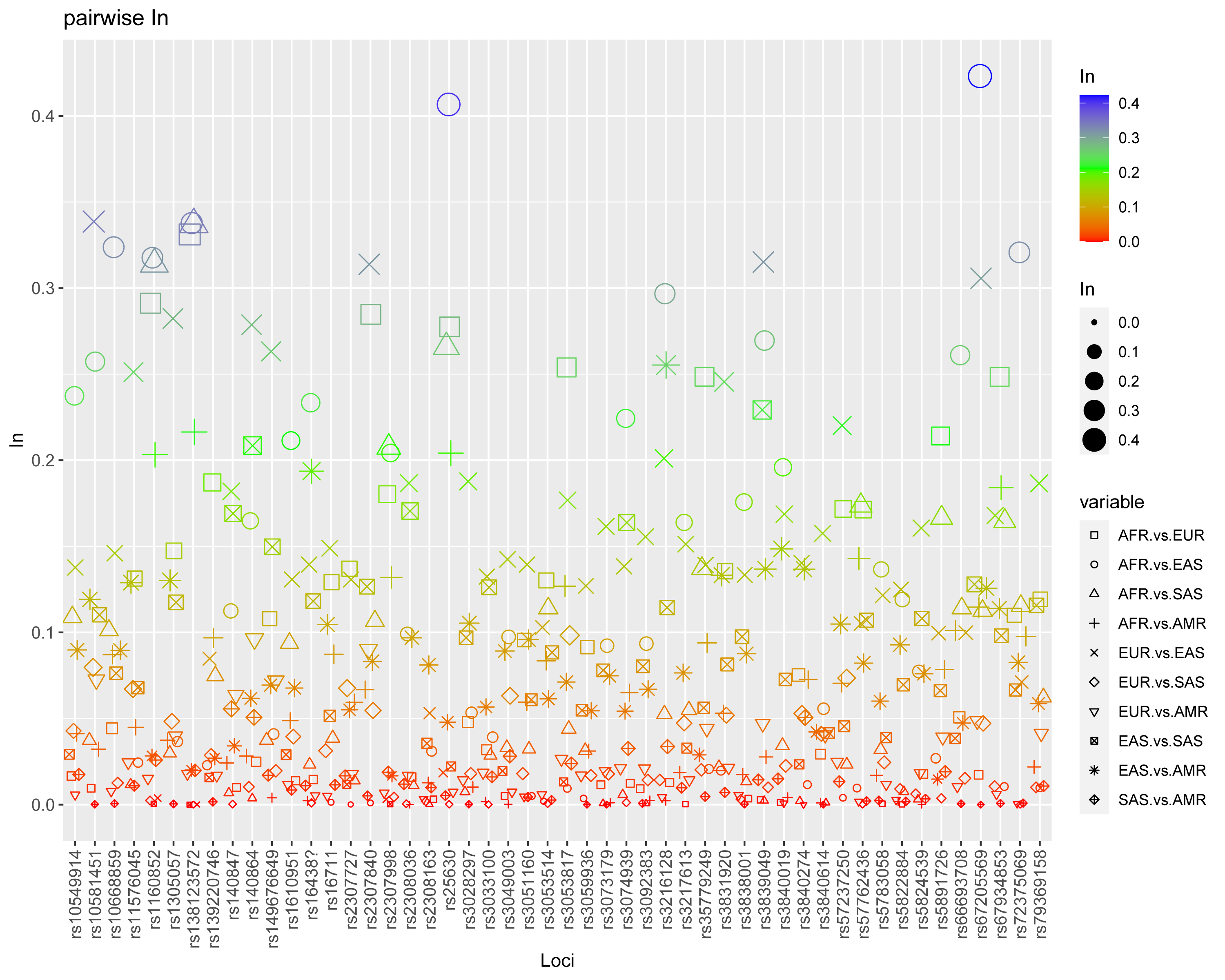

Supplement: Supplementary file 4 [file Image1.TIF]
